# Mammalian Pathogenesis and Transmission of Avian Influenza A(H7N9) Viruses, Tennessee, USA, 2017

**DOI:** 10.3201/eid2401.171574

**Published:** 2018-01

**Authors:** Jessica A. Belser, Nicole Brock, Xiangjie Sun, Joyce Jones, Natosha Zanders, Erin Hodges, Joanna A. Pulit-Penaloza, David Wentworth, Terrence M. Tumpey, Todd Davis, Taronna R. Maines

**Affiliations:** Centers for Disease Control and Prevention, Atlanta, Georgia, USA

**Keywords:** mammalian pathogenesis, transmission, influenza, influenza virus, viruses, H7N9 subtype, highly pathogenic avian influenza virus, low pathogenicity avian influenza virus, HPAI, LPAI, respiratory infections, outbreaks, mice, ferrets, transmission, cell culture, Tennessee, United States

## Abstract

Infections with low pathogenicity and highly pathogenic avian influenza A(H7N9) viruses affected poultry in 4 states in the southeastern United States in 2017. We evaluated pathogenicity and transmission of representative viruses in mouse and ferret models and examined replication kinetics in human respiratory tract cells. These viruses can cause respiratory infections in mammalian models.

Influenza A viruses have been associated with sporadic influenza outbreaks in commercial poultry throughout North America, typically due to low pathogenic avian influenza (LPAI) H5 and H7 subtype viruses (*1*). Recent emergence and spread of highly pathogenic avian influenza (HPAI) H7N3 and H7N8 subtype viruses in North America have underscored the capability of LPAI viruses to mutate into HPAI viruses and cause devastating losses to domestic poultry (*2*). Spread of avian influenza viruses in waterfowl flyways in North America, especially those over areas of dense commercial poultry operations, necessitates constant surveillance and study (*3*). Because these viruses are reportable to the World Organisation for Animal Health, detection of these subtypes also has a major role in trade of commercial poultry products (*4*).

## The Study

In March 2017, outbreaks of infection with HPAI H7N9 subtype virus were reported on 2 commercial broiler breeder farms in Lincoln County, Tennessee, USA. LPAI H7N9 subtype virus was concurrently and subsequently reported in commercial and backyard producer farms in Tennessee, Alabama, Kentucky, and Georgia (*5*,*6*). More than 270,000 birds died or were culled; no human cases were reported. Similar to previous epornitics of LPAI H7N9 subtype virus in Kentucky, Minnesota, and Nebraska in recent years, viruses isolated in Tennessee in 2017 were of the North American wild bird lineage and genetically and phenotypically distinct from the Asian lineage of avian influenza A(H7N9) virus circulating in China (*6*,*7*). 

This study was approved by the Institutional Animal Care and Use Committee of the Centers for Disease Control and Prevention. We examined in 2 mammalian models the pathogenicity and transmissibility of LPAI and HPAI H7N9 subtype viruses isolated from chickens in Tennessee (ck/TN) and evaluated the capacity for these viruses to replicate in a representative human respiratory cell line. H7N9 isolates A/ck/TN/17-007147-2/2017 (HPAI) and A/ck/TN/17-007431-3/2017 (LPAI) differ by a 9-aa insertion in the hemagglutinin gene and 18 additional amino acids throughout the genome (*5*,*6*).

Previous investigations of H7 subtype virus pairs displaying varied pathogenicity in poultry have shown differential phenotypes in mice, ranging from mild to moderate infection, which was indistinguishable between HPAI and LPAI viruses (H7N3 subtype isolates from Chile in 2002 and British Columbia, Canada, in 2004), and severe infection with HPAI, but not LPAI, viruses (H7N8 subtype isolates from Indiana, USA, in 2016) (*8*,*9*). Among LPAI or HPAI ck/TN viruses, inoculated BALB/c mice showed mild illness (weight loss <3%) and no deaths (Table). Although LPAI virus replicated to moderate titers in lungs of mice after high-dose inoculation, HPAI virus was not detected. Infectious virus was not detected in the nose or brain of any mouse.

**Table:** Infection of mice and ferrets with influenza A(H7N9) ck/TN viruses, Tennessee, USA, 2017*

Characteristic	LPAI	HPAI
Mice†		
Weight loss‡	1.7	2.3
Virus titer§		
10^6^ lung, day 3 pi	4.3 ± 1.1	<1.5
10^3^ lung, day 3 pi	2.8 (1/3)	<1.5
10^6^ lung, day 6 pi	5.6 ± 2.1	<1.5
10^3^ lung, day 6 pi	<1.5	<1.5
Ferrets, days 1–10 pi¶		
Weight loss‡	3.1	4.6
Fever#	1.2	0.6
Virus in rectal swab specimen**	2/3	1/3
Virus titer at day 3 pi§		
Nasal wash	4.9 ± 0.5	3.6 ± 0.9
Nasal turbinates	5.8 ± 0.6	4.8 ± 0.8
Trachea	3.5 ± 0.4 (2/3)	<1.5
Lung	<1.5	<1.5
Olfactory bulb	3.8 (1/3)	<1.5
Brain	<1.5	<1.5
Intestine	<1.5	<1.5

*EID_50_, 50% egg infectious dose; HPAI, highly pathogenic avian influenza virus; LPAI, low pathogenicity avian influenza virus; pi, postinoculation. †Mice were 8 weeks of age and inoculated intranasally with 10^6^ EID_50_ or 10^3^ EID_50_ of virus in a volume of 50 μL. ‡Percent mean maximum weight loss after inoculation with 10^6^ EID_50_ of virus (days 2–10 pi). §Virus titers are expressed as mean ± SD log_10_ EID_50_/mL for animals with positive virus detection (n = 3 unless otherwise denoted in parentheses). Inoculation dose was 10^6^ EID_50_ (mice and ferrets) or 10^3^ EID_50_ (mice). The limit of virus detection was 10^1.5^ EID_50_/mL. ¶Ferrets were 7 mo of age, serologically negative for currently circulating viruses by hemagglutinin inhibition assay, and inoculated intranasally with 1 mL of virus. #Mean maximum increase in body temperature (baseline body temperature range 38.1°C–38.9°C). **No. ferrets with detectable virus in rectal swab specimens collected on days 1, 3, and 5 pi/total no. ferrets tested.

To examine virulence of ck/TN viruses in a mammalian species with closer physiologic similarity to humans, ferrets were inoculated with LPAI or HPAI viruses. Both viruses caused mild infection (<5% mean maximum weight loss; Table) without sustained lethargy, sneezing/nasal discharge, or high fever. LPAI and HPAI viruses were restricted to the upper respiratory tract of ferrets, and no infectious virus was detected in lungs. Both viruses were detected in nasal turbinates of all inoculated ferrets, and LPAI virus was also detected in the trachea of 2/3 ferrets and the olfactory bulb of 1/3 ferrets. Low virus titers were detected in rectal swab specimens (Table) but not in intestinal tissue or ocular samples (conjunctival washes and eye and conjunctiva tissue). These findings indicate that HPAI and LPAI ck/TN viruses have mild virulence for 2 mammalian species.

**Figure 1:**
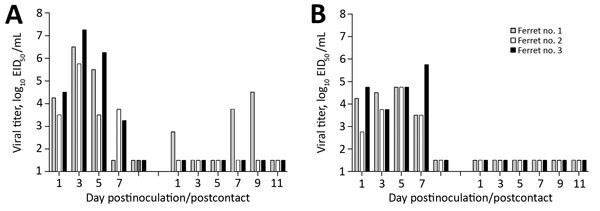
Transmission of avian influenza A(H7N9) virus ck/TN between ferrets in direct contact, Tennessee, USA, 2017. Three ferrets were inoculated with 106 EID50 of A) low pathogenicity avian influenza virus or B) highly pathogenic avian influenza virus, and nasal washes were collected from each ferret on the indicated days postinoculation (left bars) to assess viral replication. An immunologically naive ferret was placed in the same cage as each inoculated ferret at 24 h postinoculation, and nasal washes were collected from each contact ferret on the indicated days postcontact. Bars indicate individual ferrets. All ferrets were serologically negative for circulating influenza viruses at the start of the study. The limit of virus detection was 101.5 EID50/mL. EID50, 50% egg infectious dose.

Selected HPAI and LPAI H7 subtype influenza viruses from North America have the capacity to be transmitted between ferrets when the animals are placed in close contact (*10*). However, the transmissibility of ck/TN H7N9 viruses was not known. Ferrets inoculated with LPAI and HPAI ck/TN viruses shed virus in nasal wash specimens through days 5–7 postinfection and showed seroconversion to homologous virus (Figure 1). HPAI virus was not transmitted to immunologically naive cage mates because all contact ferrets remained seronegative at the end of the study. However, 1/3 LPAI virus contact ferrets shed virus to titers >10^4^ 50% egg infectious dose/mL and seroconverted to homologous virus, which demonstrated a limited capacity for virus transmission in this model.

**Figure 2:**
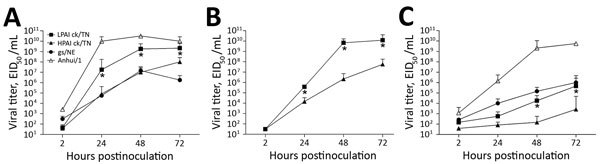
Replication kinetics of avian influenza A(H7N9) viruses in human respiratory tract cells, Tennessee, USA, 2017, compared with strains from Nebraska (gs/NE) and Asia (Anhui/1). Calu-3 cells (American Type Culture Collection, Manassas, VA, USA) were grown to confluence in 12-mm–diameter transwell inserts (Corning, Corning, NY, USA), infected apically with viruses shown at a multiplicity of infection of 0.01 (A and C) or 0.001 (B) 1 h, washed, and incubated at 37°C (A and B) or 33°C (C). Supernatants were removed at indicated times postinoculation, and titers of infectious virus were determined by titration in eggs. The limit of virus detection was 101.5 EID50/mL. Values are mean from triplicate independent cultures per virus. Error bars indicate SDs. *p<0.05 for HPAI vs. LPAI ck/TN viruses by 2-way analysis of variance with a Tukey posttest. EID50, 50% egg infectious dose; gs, goose; HPAI, highly pathogenic avian influenza virus; LPAI, low pathogenicity avian influenza virus.

To determine if the growth advantage of LPAI virus in mice and ferrets was maintained in human cells, we compared replication kinetics of HPAI and LPAI ck/TN viruses in a human bronchial epithelial cell line (Calu-3). Although both viruses replicated to high titers (>10^7^ 50% egg infectious dose/mL) at a multiplicity of infection of 0.01 or 0.001 (Figure 2, panels A, B), LPAI virus replicated to a significantly higher titer than HPAI virus at either multiplicity of infection or culture temperature tested (p<0.05; Figure 2), despite showing a temperature sensitivity at 33°C, which was similar to that for other avian influenza viruses (*11*). Virus titers for LPAI ck/TN virus were significantly higher than those for a previously studied LPAI H7N9 virus from North America (A/goose/Nebraska/17097-4/2011) 24–72 h postinfection at 37°C (p<0.05), but were reduced compared with the Asian lineage H7N9 subtype A/Anhui/1/2013 virus (Figure 2, panel A).

## Conclusions

Detection of HPAI and LPAI H7 viruses in the United States represents a threat to commercial poultry activities and avian health. Limited transmission in a direct contact setting of LPAI (but not HPAI) ck/TN virus is similar to virus transmission of LPAI H7N8 and H7N2 subtype viruses (*8*,*10*), and suggests that influenza A(H7N9) viruses isolated in the United States in 2017 represent a low threat for human health in their current form. However, the ability of H7 subtype viruses in North America to acquire genetic insertions at the hemagglutinin cleavage site after recombination with host RNA (*12*,*13*) and their potential to acquire mutations associated with mammalian adaptation and virulence underscores the need to monitor birds for LPAI H7 viruses. This monitoring is especially needed in and around regions with wild bird flyways and high density of poultry because these viruses can rapidly and sporadically mutate to become HPAI viruses (*3*).
